# Detection of DOPA-Melanin in the Dimorphic Fungal Pathogen *Penicillium marneffei* and Its Effect on Macrophage Phagocytosis In Vitro

**DOI:** 10.1371/journal.pone.0092610

**Published:** 2014-03-19

**Authors:** Donghua Liu, Lili Wei, Ting Guo, Weifen Tan

**Affiliations:** Department of Dermatology and Venereology, First Affiliated Hospital, Guangxi Medical University, Nanning, China; King's College London Dental Institute, United Kingdom

## Abstract

The fungal pathogen *Penicillium marneffei* produces melanin-like pigment *in vitro*. The synthetic pathway of melanin and its possible influence in the protective yeast cells surviving within macrophage cells are not known. In this work, *P. marneffei* produced brown black pigment in the presence of L-DOPA and black particles were extracted from yeast cells treated with proteolytic enzymes, denaturant and concentrated hot acid. Kojic acid inhibited the brown-black pigment production of *P. marneffei* yeast grown on brain heart infusion agar. Transmitting electron microscopy showed spherical granular electron-dense particles with an average diameter of 100 nm in a beaded arrangement in the innermost cell wall. Electron-paramagnetic resonance revealed that the black particles contain a stable free radical compound. The UV-visible and Fourier transform infrared spectra of particles extracted from *P. marneffei* and synthetic DOPA-melanin showed a high degree of similarity. Melanized yeast cells decreased phagocytosis by macrophage cells and increased resistance to intracellular digestion *in vitro*. These results indicate that *P. marneffei* can synthesize DOPA-melanin or melanin-like compounds *in vitro* and suggest that the DOPA-melanin pathway is associated with cell wall structure and enhances the resistance to phagocytosis by macrophages.

## Introduction

*Penicillium marneffei*, recently renamed as *Talaromyces marneffei* [1], is a thermally dimorphic fungus that appears in mycelia form at 25°C and yeast form at 37°C. The fungus is endemic in Southeast Asia and causes systemic infection in humans, especially in immune suppressed individuals. *P. marneffei* infection is fatal if untreated in immunocompromised hosts. The pathogen has become one of the most common HIV-related infections in Southeast Asia [2], [3].

Once *P. marneffei* invades the host, it proliferates first in the reticuloendothelial system before disseminating throughout the host. The ability of *P. marneffei* yeast cells to survive within host cells is essential to enable the fungus to produce systemic infection, and this survival ability renders the fungus difficult to be completely cleared from the body. The mechanisms by which *P. marneffei* protects itself from the host immune defense remain unclear. Expression of acid phosphatase activity in *P. marneffei* yeast cells [4], and the upregulation of the *cpeA* and *sodA* genes in this yeast phase [5], [6], may be associated with the oxidative stress response during intracellular infection.

Melanin pigments are dark brown and black pigments formed by oxidative polymerization of phenolic compounds. These high molecular weight amorphous polymers are widely found in bacteria, fungi, plants and animals. Many fungi synthesize melanins, and several types of melanins are known to exist in the fungal kingdom. Two major types of melanin, 1,8-dihydroxynaphthalene (DHN) and L-3,4-dihydroxyphenylalanine (DOPA), are found in fungi. Fungal melanin contributes to virulence in an array of human pathogen fungi, including *Cryptococcus neoformans* [7], [8], *Paracoccidioides brasiliensis* [9], *Aspergillus fumigatus* [10], *Histoplasma capsulatum* [11], *Blastomyces dermatitidis* [12] and *Malassezia furfur* [13]. Fungal melanin can influence the immune response of the host by several pathways, including reduction of the oxidative burst capacity of macrophages [14], inhibition of apoptosis in macrophages [10], and inhibition of cytokine production in the host [15].

*P. marneffei* conidia and yeast cells can produce melanin or melanin-like compounds *in vitro* and *in vivo* [16]. The polyketide synthase gene of the melanin-biosynthesis gene cluster (alb1) in *P. marneffei* contributes to melanin pigment production and the pathogen virulence [17]. Whether *P. marneffei* synthesizes melanin via the DHN pathway, DOPA pathway, or both pathways has not been determined.

The distribution of melanin in *P. marneffei* yeast cells and the role of melanin during intracellular infection remain unclear. There are two possible starting molecules in the DOPA pathway of melanin synthesis, L-DOPA or tyrosine. In the present study, we examined the role of L-DOPA on the synthesis of melanin in *P. marneffei* and the physicochemical properties of melanin isolation from *P. marneffei* yeast cells. Given the potential role of melanin in the virulence of *P. marneffei*, we also investigated the effect of melanin in yeast cells on their phagocytosis by Ana-1 mouse macrophage cells.

## Materials and Methods

### Fungal strain and preparation of inocula

The *P. marneffei* FRR2161 strain was generously donated by Dr. Alex Andronopolous (Melbourne University, Australia). The GXMU110608 *P. marneffei* strain was isolated from an HIV positive patient through regular fungal culture for diagnosis at the first affiliated hospital of Guangxi Medical University in 2006. Stock cultures were maintained in our laboratory with 6-month transfers to potato dextrose agar (PDA). To obtain the yeast phase of *P. marneffei*, colonies grown on PDA at 25°C were transferred to culture on brain heart infusion agar (BHI) at 37°C and maintained by continuous weekly passages until 70% or more cells transformed into yeast-like cells. The cells were harvested with 0.1% Tween80 and the number of fungi was counted with a hemocytometer. A conidia suspension that contained approximately 1–5×10^6^ cell per milliliter was used as the inoculum for shake culture.

### L-DOPA culture

*P. marneffei* FRR2161 and GXMU110608 yeast cultures were grown on a chemically defined liquid medium (0.22 M glucose, 2.0 mM MgSO_4_.7H_2_O, 1.8 mM KH2PO4, 15.2 mM asparagine, and 1.7 mM vitamin B1) with or without 1.0 mM L-DOPA (Sigma Chemical Co., U.S.) for 15 days at 37°C in a rotary shaker at 200 rpm. The chemically defined liquid medium with 1.0 mM DOPA but no *P. marneffei* was designated as control. All cultures were incubated in the dark to avoid photo polymerization of L-DOPA into melanin. Yeast cells were smeared and stained by cotton blue, and then samples were examined under optical microscope. Cells were collected by centrifugation at 2000 rpm for 30 min, washed with PBS, and stored at 4°C until use.

### Culture with or without melanin inhibitors

Kojic acid and tricyclazole have been used as inhibitors to study the pathway of melanin synthesis in a few fungal species [13], [18], [19]. To investigate the melanin pathway in *P. marneffei*, FRR2161 and GXMU110608 strains were grown on BHI slant culture at 37°C. An inoculation loop was used to transfer yeast cells from slant culture to BHI plates, BHI plates with 100 μg/ml kojic acid, or BHI plates with 100µg/ml tricyclazole (Sigma Chemical Co., U.S.). A BHI plate with 1.0 mM DOPA but no *P. marneffei* was designated as control. After culture at 37°C for 7 days, the color of colonies was observed.

### Electron microscopy

The culture suspensions of FRR2161 and GXMU110608 strains from chemically defined liquid medium were placed in 3% glutaraldehyde and fixed overnight at 4°C. Samples were then applied to a polylysine-coated coverslip, serially dehydrated in alcohol and replaced with isoamyl acetate for further dehydration. Samples were then dried by the critical-point drying method and further evaporated with carbon. The sample was coated with gold-palladium and the surface structures of yeast cells were observed by a scanning electron microscope (JSM-T300, Japan). For transmission electron microscopic observation, the culture suspension was allowed to stand for 1 h, and the precipitate was embedded in 2% agar and then fixed overnight in 3% glutaraldehyde at 4°C. The sample was incubated in 1% osmic acid for 2 h, dehydrated, and embedded in epoxy resin. Ultrathin sections were placed on nickel grids and examined using a Hitachi H-500 transmission electron microscope.

### Isolation and purification of yeast melanin particles

Melanin particles were isolated from FRR2161 and GXMU110608 strains grown on chemically defined liquid medium supplemented with 1.0 mM L-DOPA. Yeast cells were collected by centrifugation at 5000 rpm for 10 min. The pellet was washed three times with PBS, and suspended in 1.0 M sorbitol/0.1 M sodium citrate (pH 5.5). Cells were collected by centrifugation, wall-lysing enzymes (from *Trichoderma harzianum* [Sigma-Aldrich]) dissolved in doubled distilled water were added at 15 mg/ml, and the suspension was incubated overnight at 30°C to generate protoplasts. The protoplasts were collected by centrifugation, washed with PBS, and incubated in 4.0 M guanidine thiocyanate overnight at room temperature. Cell debris were collected by centrifugation, washed three times with PBS, and digested with 1.0 mg/ml proteinase K overnight at 37°C. The resulting product was washed three times with PBS and then boiled in 6.0 M HCl for 1.5 h. The insoluble residue was collected by centrifugation, washed extensively with distilled water, and dried to obtain a black pigment crude product. Black pigment crude product was fully dissolved in 1 M NaOH; the supernatant was collected by centrifugation, adjusted with 6 M HCI to pH 2–3, allowed to stand overnight, and the precipitate was collected by centrifugation. The procedure was repeated three times. The precipitate was washed extensively with distilled water and dried to obtain a black pigment purified product.

### Characterization of melanin pigment

The solubility of purified *P. marneffei* pigment from FRR2161 and GXMU110608 strains and synthetic DOPA-melanin (2 mg) were tested with distilled water, hot 1.0 M NaOH solution and common organic solvents (methanol, absolute ethanol, propanol, acetone, acetonitrile, iso-amyl alcohol, and chloroform). Reactions with oxidizing agents such as 30% hydrogen peroxide (H_2_O_2_) were determined. Purified *P. marneffei* melanin was subjected to various analyses such as UV-visible absorption (Varian Cary50), Fourier transform infrared (FT-IR) spectra (Perkin Elmer Spectrum100) and electron-paramagnetic resonance (EPR) (Bruker EMX-10/12). The UV absorption was performed using 40 μg/ml *P. marneffei* melanin dissolved in sodium hydroxide scanning at approximately 200–800 nm wavelength. The dried 2 mg *P. marneffei* melanin was mixed with 200 mg potassium bromide by grinding in an agate mortar for 5 min, and the sample was examined by infrared spectral scanning at approximately 4000–400 cm^-1^. The various instrumental parameters of EPR were set at 100 kHZ modulation frequency, 1.0 G modulation amplitude, 5.0 mW microwave power, 9.75 GHZ microwave frequency, 3475.0 G center magnetic field strength, 100.0 G scan width and 83.89 s scan time.

### In vitro phagocytosis

The FRR2161 strain was inoculated in BHI slants at 37°C, and generations were transferred every three days until pure yeast cells were acquired. The *P. marneffei* colonies were flooded with PBS and the number of fungi was counted with a hemocytometer after washing. The concentration of yeast suspension was adjusted to 1×10^7^/ml for experimental use. Ana-1 mouse macrophage cells (1×10^6^/bottle) were cultured in RPMI-1640 medium containing 10% fetal bovine serum. Yeast cells in a 200 μl suspension were cultured with the Ana-1 mouse macrophage cell line pretreated with 1 μg/ml recombinant murine IFN-γ. Culture groups were divided into the experimental group cultured with 1 mM L-DOPA and the control group cultured without L-DOPA. Ana-1 mouse macrophage cells cultured with 1 mM L-DOPA without yeast cells were designated as blank controls. The macrophage cells and yeast cells were observed under an inverted microscope during the 7 days of culture, after which cells were collected by centrifugation at 3000 rpm for 5 min. The method of sample preparation for transmission electron microscopy was described as above. The tests were repeated three times on different days.

### Statistical analysis

The average number of yeast cells per macrophage in *P. marneffei* cultured with L-DOPA was compared to control. The data was analyzed by t-test for two independent samples, with significance defined as *P*<0.05.

## Results

### Colors of medium and isolation from culture

**Figure 1:**
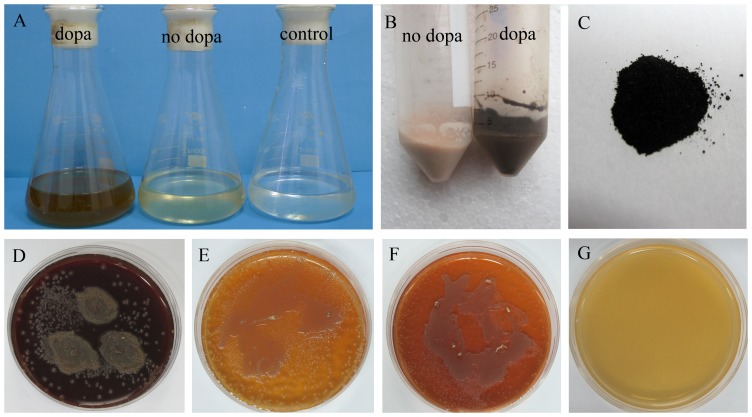
Black pigment production of *P. marneffei* in media under various culture conditions. FRR2161 strain grown on chemically defined liquid medium with or without L-DOPA, control flask (A) and the yeast cells isolated from the medium (B). Black particles isolated from the dark brown yeast cells(C). FRR2161 strain grown on BHI (D), BHI plates with 100 μg/ml kojic acid (E) or BHI plates with 100 μg/ml tricyclazole (F) and a BHI plate with DOPA but no *P. marneffei*(G).

The liquid medium colors of the cultured FRR2161 and GXMU110608 strains supplemented with L-DOPA at 37°C by shake culture were brown-black. No pigmentations were observed in *P. marneffei* strains cultured without L-DOPA and in control medium (Fig. 1A). Pigment production was not observed in control flasks, indicating that L-DOPA autopolymerization does not occur. The yeast cells of *P. marneffei* isolated from the chemically defined medium with L-DOPA appeared as dark brown (Fig. 1B) and darkly pigmented particles was isolated from the yeast cells (Fig. 1C). When cultured on BHI at 37°C, the yeast colony surface was smooth, its color was black/tan, and brown-black pigment was produced around the colony (Fig. 1D). However, when cultured on BHI at 37°C with kojic acid (Fig. 1E) or tricyclazole (Fig. 1F), the color of the yeast colony became lighter, and no brown-black pigment was produced around the colony cultured with kojic acid. Pigment production was also not observed in control plate (Fig.1G).

### Morphological analysis by electron microscopy

**Figure 2:**
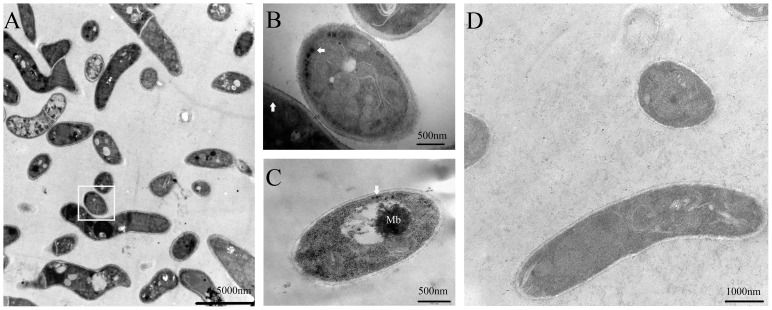
TEM examination of *P. marneffei* FRR2161 cultured in chemically defined liquid medium with or without L-DOPA. Spherical granular electron-dense particles (white arrowheads) were in a beaded arrangement in the innermost cell wall in the presence of L-DOPA (A, B, C).Magnification of area indicated by rectangle in panel A (B). Enlargement of microbody (Mb) size with electron-dense material increased was observed(C). No spherical granular electron-dense particles were observed in yeast cells without L-DOPA (D).

Yeast cells grown with or without L-DOPA showed no difference as observed by scanning electron microscopy. Transmission electron microscopy analysis of yeast cells supplemented with L-DOPA showed spherical granular electron-dense particles with an average diameter of 100 nm in a beaded arrangement in the innermost cell wall (Fig. 2A, B, C). Enlargement of microbodies size with electron-dense material increased significantly was observed (Fig. 2C). The other cell organelles, such as mitochondrion and liposome body, had no significant changes. Spherical granular electron-dense particles and electron-dense material deposited in microbodies were not seen in the yeast cells cultured without L-DOPA (Fig. 2D).

### Physicochemical analysis of *P. marneffei* melanin

**Figure 3:**
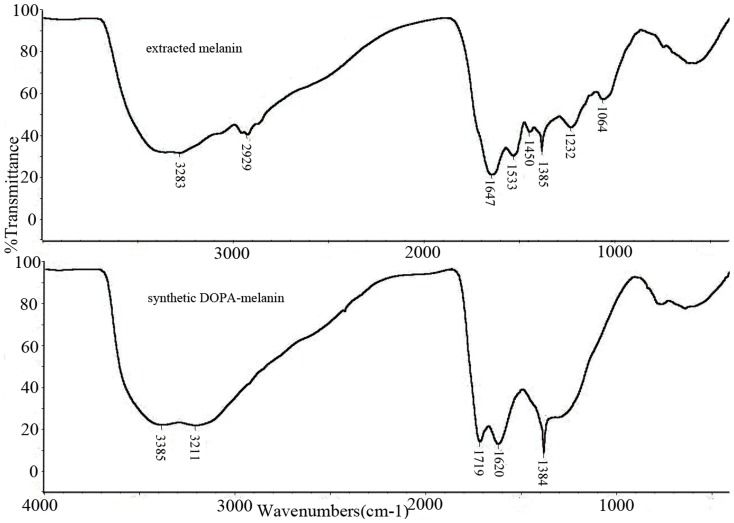
FT-IR analysis of the melanin extracted from *P. marneffei* and the synthetic DOPA-melanin.

**Figure 4:**
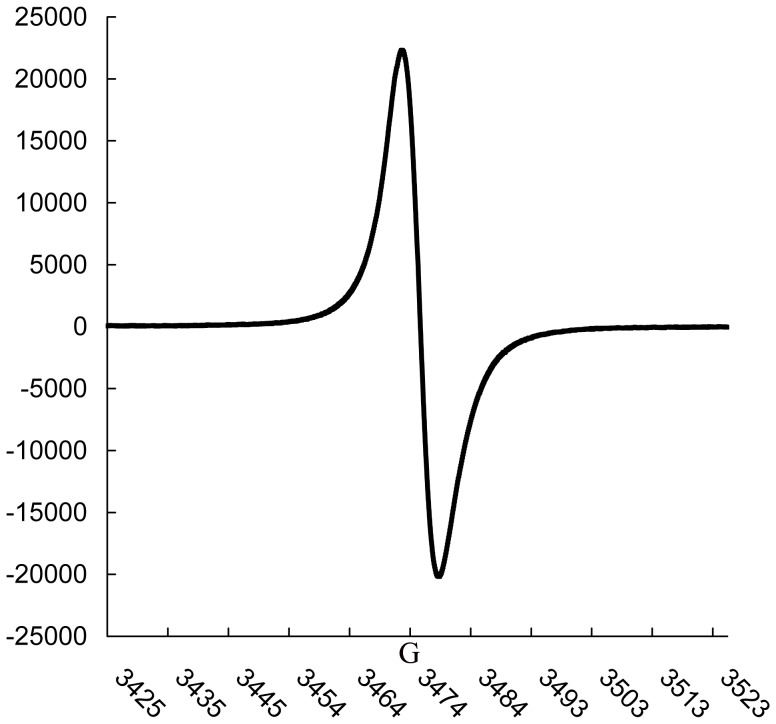
EPR spectral analysis of the black particles extracted from *P. marneffei*.

The physicochemical properties of the melanin from *P. marneffei* were very similar to synthetic DOPA-melanin. Both substances were insoluble in water and common organic solvents, but were soluble in hot NaOH; they precipitated in concentrated HCl and decolorized in the presence of H_2_O_2_. These characteristics are common to various melanin described in literature [20], [21], [22]. UV-vis analysis indicated that melanin extracted from *P. marneffei* had a typical absorption peak at 205 nm and absorbed progressively less as the wavelength increased. In the FT-IR, the sample showed a broad absorption at 3283 cm^-1^, indicating the presence of an OH group and NH_2_ group. Absorption at 1647 cm^-1^ was attributed to aromatic ring C = C stretching spectra. The extracted melanin and synthetic DOPA-melanin showed similar spectrum (Fig. 3). However, the absorption peaks of extracted melanin had several different peaks compared with synthetic DOPA-melanin: (i) the peak occurred at 2929 and 1450 cm^-1^, which indicated a CH2 group and CH3 group, respectively, and (ii) the peaks of 1533, 1385, 1232 and 1064 cm^-1^ indicated N-H, C-N, C-OH and C-O stretching spectra, respectively. EPR spectroscopy is a particularly effective method for studying melanin pigments, since these pigments uniquely contain a stable population of organic free radicals [23]. In this study, EPR revealed that particles from the yeast cells of *P. marneffei* contained a stable free radical compound (Fig.4). The presence of stable paramagnetic species is a characteristic of the melanin pigment [23].

### Effect of DOPA-melanin on phagocytosis

**Figure 5:**
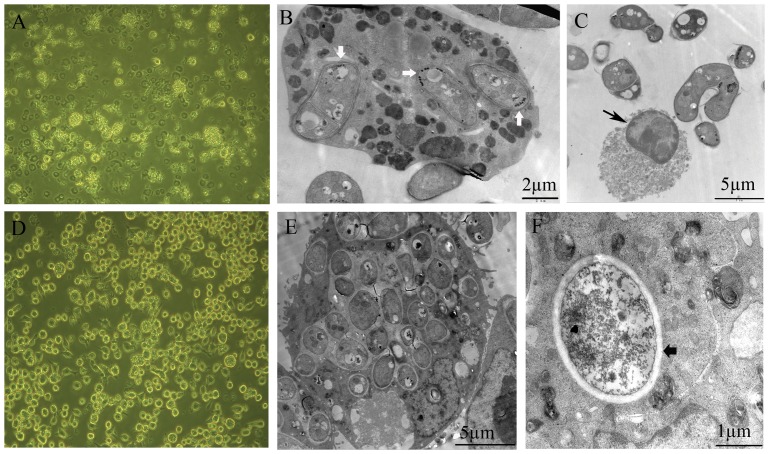
Microscopic examination of macrophages cultured with *P. marneffei*. Culture of Ana-1 mouse macrophage cells with *P. marneffei* in the presence of L-DOPA (A:×200). Phagocytosis of the melanized yeast cells (short white arrowheads) by Ana-1 mouse macrophage cells (B), disintegration of macrophage cells (black arrowhead) and the extracellular melanized yeast cells (C). Culture of Ana-1 mouse macrophage cells with *P. marneffei* without L-DOPA (D:×200). Phagocytosis of the non-melanized yeast cells by Ana-1 mouse macrophage cells (E), and intracellular digestion of non-melanized yeast cells (short black arrowhead) (F).

The number of macrophage cells infected with *P. marneffei* yeast cells in the presence of L-DOPA decreased compared to the control culture infected with *P. marneffei* yeast cells without L-DOPA after 7 days of culture as observed by an inverted microscope (Fig.5A, D). The number of macrophage cells in the blank control (culture without yeast cells in the presence of L-DOPA) increased with normal cell morphology. Transmission electron microscopy was used to analyze cells, and yeast cells with the beaded arrangement of spherical granular electron-dense particles were defined as melanized cells. Under transmission electron microscopy, phagocytosis of 1–18 melanized yeast cells by a single macrophage cell was observed, with an average 6.5±4.3 (mean±SD) yeast cells per macrophage in an examination of 100 macrophages (Fig. 5B). The yeast cells with the beaded arrangement of spherical granular electron-dense particles in the inner cell wall maintained cell structural integrity within macrophage cells. Disintegration of macrophage cells was observed in the group infected with melanized yeast cells (Fig. 5C). Phagocytosis of 5–25 non-melanized yeast cells by a single macrophage cell was observed, with an average 11.6±4.4 (mean±SD) yeast cells per macrophage in an examination of 100 macrophages (Fig. 5E). Intracellular digestion of non-melanized yeast cell was observed (Fig. 5F), but the frequency was low. The average number of yeast cells per macrophage in the group in the presence of L-DOPA was significantly less than that of the control group (*P* < 0.01).

## Discussion

Several fungi, such as *C. neoformans* [24] and *P. brasiliensis* [25], synthesize melanin via the DOPA pathway in a process resembling mammalian melanin biosynthesis. There are two possible starting molecules in this pathway, either L-DOPA or tyrosine. In this study, we found that *P. marneffei* can synthesize DOPA-melanin or melanin-like compounds *in vitro*. Our evidence supporting the DOPA-melanin production of *P. marneffei* yeast is as follows: (i) darkly pigmented liquid medium of *P. marneffei* culture in the presence of L-DOPA, (ii) spherical granular electron-dense particle beaded arrangement at the inner cell wall observed by transmission electron microscopic in yeast cells cultured both in defined liquid medium and in RPMI-1640 medium supplemented with L-DOPA; (iii) isolation of black particles from yeast cells of *P. marneffei* cultured in the presence of L-DOPA; (iv) EPR demonstration of particles from the yeast cells of *P. marneffei* containing a stable free radical compound; (v) particles extracted from *P. marneffei* and synthetic DOPA-melanin showed similar physicochemical characterization and spectra analysis by UV-vis and FT-IR; and (vi) kojic acid inhibition of the brown-black pigment production of *P. marneffei* grown on BHI. Kojic acid effectively inhibits the rate of formation of pigmented product due to the direct effect of kojic acid on tyrosinase [26], [27]. Kojic acid significantly reduced the production of DOPA-melanin or melanin-like compounds in *Malassezia furfur* [13] and *Klebsiella* sp. GSK [22]. Kojic acid effectively inhibited the black pigment production of *P. marneffei* grown on BHI, indicating the yeast cells produce DOPA-melanin or melanin-like compounds. In the present study, we also observed that tricyclazole inhibited the brown-black pigment production of *P. marneffei* grown on BHI at 37°C, and a previous study confirmed that the PKS gene cluster regulates the melanin pigment production [17]. These results indicate that *P. marneffei* can synthesize DHN-melanin *in vitro*. All evidence supports *P. marneffei* synthesis of melanin by both the DOPA pathway and DHN pathway *in vitro*. It is noteworthy that both melanin pathways are found in another dimorphic fungus *Sporothrix schenckii* [28]. The observation that fungi have both DOPA and DHN pathways for melanin production may be associated with a concomitant increase in protection against unfavorable conditions in the environment and during infection.

Fungal melanin is associated with cell wall structure. Solid-state nuclear magnetic resonance techniques were used to track the process of melanin synthesis in *C. neoformans*, and results suggested that melanin may act as a cell wall component attached to polysaccharide [29]. The arrangement of melanin in the cell wall is dependent on the fungal species. Melanin in *C. neoformans* was found deposited in the innermost layer of the cell wall and composed of granular particles approximately 40–130 nm in diameter, and the melanin was synthesized at sites of vesicles associated with lipid [30], [31], [32]. In contrast, in *Histoplasma capsulatum*, melanin was found on the outer layer, and yeast cells grown with L-DOPA formed tufts on the cell surface [11]. Similarly, *Fonsecaea pedrosoi* showed patched granules with an average diameter of 47 nm on the cell surface [19]. However, melanin in *P. brasiliensis* was found at the cell surface and cytoplasm [9]. In this study, DOPA-melanin of *P. marneffei* composed of spherical granular particles approximately 100 nm in diameter were found in the innermost layer of the cell wall, and the melanin particles were in a beaded arrangement. The beaded arrangement of melanin particles has not been previously found in other fungi, and thus may be a characteristic feature for DOPA-melanin of *P. marneffei.* Recent evidence supports the hypothesis that fungal melanosomes exist. In *Fonsecaea pedrosoi*, melanin is stored in intracellular vesicles, named melanosomes. The melanosome fuses with the fungal cell membrane, where the melanin is released and reaches the cell wall [33]. The ultrastructure of *P. marneffei* cells grown on PDA or BHI palates without L-DOPA, such as mitochondria, endoplastic reticulum, liposome bodies and microbodies were observed by TEM in our previous study [34], no beaded electron-dense particles were observed. The beaded structure of *P. marneffei* cells in the presence of L-DOPA may be a melanosome of this yeast pathogen. Aliphatic groups (CH2 group and CH3 group) in the melanin from *P. marneffei* suggest that melanin synthesis may have an association with yeast cell wall components, such as polysaccharide attachment to melanin. The enlarged microbodies and electron-dense material increased significantly when the yeast cells of *P. marneffei* were cultured with L-DOPA. However, other cell organelles showed no significant changes. This suggests that the microbodies may be the synthesis site of melanin.

In certain fungal species, melanized cells of fungi inhibit phagocytosis by macrophages and decrease susceptibility to killing by free radicals or hydrolytic enzymes [9], [14], [35]. The average number of yeast cells per Ana-1 mouse macrophage in the presence of L-DOPA was significantly less than that of cultures without L-DOPA, which indicated that melanized *P. marneffei* inhibit phagocytosis by macrophages. Yeast cells of *P. marneffei* cultured with L-DOPA were not observed undergoing intracellular digestion by Ana-1 mouse macrophage cells, indicating that DOPA-melanin increased the resistance of yeast cells to hydrolytic enzymes. Furthermore, the reduction of the number of Ana-1 mouse macrophage cells and disintegration of macrophages in the presence of *P. marneffei* cultured with L-DOPA suggest that melanized yeast cells increase virulence to macrophages.

Taken together, the results obtained in this study indicate that *P. marneffei* can synthesize DOPA-melanin or melanin-like compounds when grown *in vitro*. The melanin particles may be associated with the cell wall structure in yeast form of *P. marneffei*. Melanized *P. marneffei* enhance the resistance to phagocytosis by macrophages and it even enhances the virulence to macrophage cells. Further studies should be performed on the genetic regulation of the DOPA pathway in the *P. marneffei* yeast form and the role of melanin as a potential target for therapeutic intervention in penicilliosis.
